# Phenolic Profile, Antiradical and Antitumour Evaluation of Raspberries Pomace Extract from Serbia

**Published:** 2017

**Authors:** Jasna Čanadanović-Brunet, Jelena Vulić, Tatjana Ćebović, Gordana Ćetković, Vladimir Čanadanović, Sonja Djilas, Vesna Tumbas Šaponjac

**Affiliations:** a *University of Novi Sad, Faculty of Technology, Bulevar Cara Lazara 1, Novi Sad, Serbia.*; b * University of Novi Sad, Faculty of Medicine, Hajduk Veljkova 3, Novi Sad, Serbia.*

**Keywords:** raspberry pomace, HPLC, antioxidant activity, antioxidant enzymes

## Abstract

Raspberry waste, obtained from two cultivars, Meeker (ERM) and Willamette (ERW) was subjected to evaluation antioxidants and antitumour activities as a potential source of phenolics. Some phenolic acids and flavonoids were identified and quantified by HPLC. Antioxidant activity was tested by measuring ability to scavenge DPPH^•^ and ^•^OH by ESR spectroscopy. IC_50_^DPPH•^ varied from 0.67 for ERM to 0.54 mg/mL for ERW, while IC_50_^•OH^ values varied from 3.73 for ERM to 1.23 mg/mL for ERW. Cytotoxic activity was investigated using *in vivo* model of Ehrlich Ascite carcinoma cells (EAC) in mice. Pretreatment with extracts exhibited potent cytotoxic activity against EAC cells (up to 60%) and both extracts inhibited the tumour growth. Activity of xanthine oxidase (XOD) was extremely increased in pretreated animals, while the activity of enzyme complex glutathione reductase (GR) and glutathione peroxidase (GSHPx) was significantly increased. This study suggests that raspberry pomace could be used as nutraceutic resource and functional food ingredient.

## Introduction

Oxidative stress, *induced by *increased levels of* free radical *species, has been associated with several cellular processes including oxidation damage to protein, lipid and DNA, that may *cause carcinogenesis and other *toxic effects* (*1*, *2*).* Therefore, intake of sufficient amounts of antioxidants is necessary *to prevent free radicals action and *oxidative stress. *A number of epidemiological studies have *consistently demonstrated that the consumption of fruits and vegetables is associated with risk reduction of cancer and other diseases. It has been suggested that the health beneﬁts of fruits and vegetables are attributed to their phytochemicals *mostly *phenolic compounds (such as ﬂavonoids and phenolic acids) *(*3*).* Studies have demonstrated that these bioactive compounds *exert their beneficial biological effects,* and hence, may promote human health through a different mechanism of action, including antioxidant activity and free radicals scavenging, inhibition of cell proliferation, induction of cell-cycle arrest, induction of apoptosis, modulation of enzyme activities in detoxiﬁcation, stimulation of the immune system as well as antibacterial and antiviral effects *(*2*, *4*).*

Raspberries (*Rubus idaeus* L.), among the most popular berries in the world, are consumed as fresh and processed to juice, jams, conﬁtures, and other products or as *ingredients for different foods* *(*5*).* *These fruits are known as a rich source* of phenolic compounds such as phenolic acids (ellagic acid as free and bound in the forms of ellagitannins and ellagic acid glycosides) and anthocyanins (cyanidin-3-sophoroside, cyanidin-3-(2G-glucosylrutinoside), cyanidin-3-rutinoside, pelargonidin-3-sophoroside, cyanidin-3-glucoside, pelargonidin-3-(2G-glucosylrutinoside), and pelargonidin-3-glucoside) *(*6*-*10*).* *Beside anthocyanins, raspberry fruits also contain *smaller quantities of other flavonoids (quercetin-3-rutinoside, quercetin-3-clucoside, quercetin-3-glucuronide and kaempferol glucuronide) *(*7*).* In addition, flavan-3-ols and their oligomers - mainly dimmers have also been *found in *raspberries *(*11*).* *It was found *that raspberry extract has one of the highest cellular antioxidant activity among *25 *commonly consumed* fruits* *(*12*).* In addition to strong antioxidant properties, other beneﬁcial bioactivities including antiinﬂammation, vasodilatory properties, antimicrobial activity against pathogenic intestinal bacteria, and antiproliferative activity on human liver, breast, colon, and prostate cancer cells, *have also been reported for* raspberries *(*3*, *6*, *7*, *13*).*

Nowdays, there is an increasing interest in ﬁnding new sources of natural bioactive compounds. The addition of bioactive compound(s) to a traditional food is one of the ways most often employed in the production of novel functional foods. The fruits and vegetables processing industries produce very large amounts of waste materials, such as skins, pulp residu,e and seeds *(*14*).* This processing waste could be a potential source of antioxidant compounds instead of being used as animal feed, fertilizer or sent to sanitary landfill *(*14*, *15*).* Based on recent studies, it is evident that different valuable compounds such as phenolics remained in the raspberry processing waste (16-19). Since fruits and vegetables processing wastes are inexpensive, easily available, and composed of bioactive molecules, it can be suggested that by-products are alternative sources of bioactive antioxidants that can contribute to a consumer's health, as a part of functional foods *(*20*-*23*).*

According to these facts, our study deals with evaluation of waste materials generated from raspberry juice processing as a potential source of bioactive antioxidants. Thus, the objectives of this study were to determine the phenolic composition and bioactive properties (antioxidant and *in-vivo* cytotoxic activity against Erlich tumour cells) of raspberry waste extracts.

## Experimental

*Chemicals*

2,2-Diphenyl-1-picrylhydrazyl (DPPH), 5,5-dimethyl-1-pyroline-*N*-oxide (DMPO), phenolic acids including gallic, protocatechuic, vanillic, ellagic, caffeic, chlorogenic, coumaric, ferulic, synapic and rosmarinic acids, quercetin, rutin, luteolin, myricetin, kaempferol, catechin, epicatechin and epicatechin gallate were purchased from Sigma Chemical Co. (St. Louis, MO, USA). These chemicals were of analytical reagent grade. Other chemicals and solvents used of the highest analytical grade were obtained from ''Zorka'' Šabac (Serbia). 

*Plant material*

Two raspberry (*Rubus idaeus *L.) cultivars (Meeker and Willamette) were obtained from “Alfa RS”, Lipolist, Serbia. Raspberries were washed in running water, cut into pieces, and raspberry pulp was prepared using a domestic food processor (Neo SK-400). After juice separation, the samples of the obtained raspberry pomace were stored at -20 °C until analysis. Moisture of each sample was determined using drying oven method, by drying a representative 10 g sample in a forced air oven (Sterimatic ST-11, Instrumentaria, Zagreb, Croatia) at 60 °C until the constant mass.

*Preparation of raspberry pomace extracts*

Samples of the wet raspberry pomaces (20 g) were extracted at room temperature using a homogenizer, Ultraturax, and DIAX 900 (Heidolph Instruments GmbH, Kelheim, Germany). The extraction was performed two times with different amounts of 80% methanol aqueous solution containing 0.05% acetic acid: 160 mL in 60 min and 80 mL in 30 min at room temperature. The obtained extracts were filtrated, combined, and evaporated to dryness under reduced pressure and lyophilisated (Alpha 2-4 LSC Martin Christ, Osterode, Germany). The yields of the lyophilisated raspberry pomace extracts were: Meeker (ERM) 2.33 ± 0.08 g and Willamette (ERW) 3.06 ± 0.09 g.

*HPLC analysis of raspberry pomaces*

All analyte solutions and solvents were filtered prior to analysis through 0.45 µm (pore size) membrane filters (Millipore, Bedford, MA). Quantification of phenolics was done by the following HPLC analysis. Samples were analyzed by a Shimadzu Prominence (Shimadzu, Kyoto, Japan) chromatographic system, which consisted of LC-20AT binary pump, CTO-20A thermostat and SIL-20A autosampler connected to the Waters SPD-20AV UV/Vis detector (Shimadzu, Kyoto, Japan). Chromatograms were recorded using different wavelength for individual compounds: 280 nm for hydroxybenzoic acids (gallic, protocatechuic, vanillic and syringic acid) and ellagic acid, 320 nm for hydroxycinnamic acids (caffeic, chlorogenic, coumaric, ferulic, synapic and rosmarinic acid) and 360 nm for flavonoids (quercetin, rutin, luteolin, myricetin, kaempferol, catechin, epicatechin and epicatechin gallate). Separation was performed on a Luna C-18 RP column, 5 µm, 250 x 4.6 mm (Phenomenex, Torrance, CA, USA) with a C18 guard column, 4 x 30 mm (Phenomenex, Torrance, CA, USA). Two mobile phases, A (acetonitrile) and B (1% formic acid) were used at flow rates of 1 mL/min with the following gradient profile: 0 - 10 min from 10 to 25% A; 10 - 20 min linear rise up to 60% A, and from 20 min to 30 min linear rise up to 70% A, followed by 10 min reverse to initial 10% A with additional 5 min of equilibration time. Reference substances (flavonoids and phenolic acids) and samples were dissolved in 50% methanol. The data acquisition were carried out by the LC Solution Software (Shimadzu, Kyoto, Japan). All analysis were run in triplicate. 

*Electron spin resonance (* *ESR) measurements*

*DPPH radical assay*

 DPPH radicals (DPPH^•^) were generated by mixing 0.2 mL water and 0.4 mL 0.4 mM methanolic solution of DPPH radicals (control) *(*24*).* The influence of extracts on the DPPH radicals was investigated by adding the water solutions of ERM (0.25 - 1.25 mg/mL) or ERW (0.1 - 1.75 mg/mL) in the reaction system. After that, the mixture was stirred for 2 min and transferred to an ER-160FT quartz flat cell. The ESR spectra were recorded on model 300E, Bruker, Rheinstetten, Germany under the following conditions: field modulation 100 kHz, modulation amplitude 0.256 G, receiver gain 5 x 10^5^, time constant 40.96 msec, conversion time 335.54 msec, center field 3440.00 G, sweep width 100.00 G, x-band frequency 9.45 GHz, power 7.96 mW, temperature 23°C. The scavenging activity (SA) value of extract for DPPH radical was defined as: SA_DPPH_^•^= 100 × (h_0_ – h_x_) / h_0_ [%]**, **where h_o_ and h_x_ are the height of the second peak in the ESR spectrum of DPPH radicals of the control and the probe, respectively.

*Hydroxyl radical* *scavenging activity*

 Hydroxyl radicals (^•^OH) were generated in the Fenton reaction system obtained by mixing 0.2 mL of 112 mM DMPO, 0.2 mL of H_2_O, 0.2 mL of 2 mM H_2_O_2_, and 0.2 mL of 0.3 mM Fe^2+^ (control) *(*25*).* The influence of extracts on the formation and stabilization of hydroxyl radicals was investigated by adding the water solutions of ERM (2.0 - 8.0 mg/mL) or ERW (0.1 – 6.0 mg/mL) in the Fenton reaction system. ESR spectra were recorded after 5 min, with the same setting as DPPH test except: modulation amplitude 0.226 G, time constant 80.72 ms, conversion time 327.68 ms, *x*-band frequency 9.64 GHz, power 20 mW. The SA^•^_OH_ value of the extract was defined as: SA^•^_OH_ = 100 × (h_0_ – h_x_) / h_0_ [%], where h_0_ and h_x_ are the height of the second peak in the ESR spectrum of DMPO-OH spin adduct of the control and the probe, respectively. 

*Cytotoxic activity against Ehrlich’s tumour in mice*

*Animals and experimental procedures* *. *Animal care and all experimental procedures were conducted in accordance with the *Guide for the Care and Use of Laboratory Animal Resources* edited by the Commission of Life Sciences, National Research Council. Male and female Hannover National Medical Institute (Hann:NMRI) mice were obtained from the Biochemical Laboratory, Clinical Centre of Vojvodina (Novi Sad, Serbia). Animals were fed standard mice chow (LM_2_, Veterinarski zavod, Subotica, Serbia) with free access to tap water, in a temperature (25 ºC) and humidity-controlled (30-50%) animal house under 12 h light/day cycles. NMRI mice of both sexes (6 - 8 weeks old), weighing 25.0 g ± 2.5 g, were used in our experiments. Animals were divided into four groups of six mice under the following conditions and treatments: I, EAC group (mice with implanted EAC cells), (n=6); II mice pre-treated with the ERM, or ERW, 2.0 mL/kg b.w. per day, i.p., starting 7 days before the EAC implantation (n=6); III, mice treated with the ERM, or ERW, 2.0 mL/kg b.w. per day, i.p., starting from the day of the EAC implantation (n=6); and IV, mice post-treated with the ERM, or ERW 2.0 mL/kg b.w. per day, i.p., starting 7 days after EAC implantation (n=6). The experiments were repeated in the same way using the 2.0 mL/kg b.w of antioxidant N-acetyl-L-cysteine (NALC) instead of the ERM, or ERW. Fourteen days after EAC implantation, all mice were anesthetized and sacrificed, and the ascites of the carcinoma were collected for further experiments. 

*Determination of tumour cell number and cell viability. *Ascites from the abdomen were transferred to a Krebs–Ringer phosphate buffer solution (0 C, pH 7.4), then subjected to subsequential centrifuging at 4500 rpm (MSE HIGH SPEED centrifuge at 4 C) and 12 000 rpm (Eppendorf 3200 centrifuge, 2.5 min) to obtain a dense cell suspension (1 : 1). The cell weight and cell number, expressed as the number of cells per mm^3^ (counted in a Neubauer's compartment), were determined from the suspension. Cell viability was determined by the Trypan blue exclusion method: Trypan blue (0.4% solution in Krebs–Ringer phosphate buffer) stained only the damaged cells. These data were expressed as a percentage of damaged cells.*Biochemical tests. *Samples were dilluted by Krebs-Ringer phosphate buffer and the activities of several antioxidant enzymes were determined in EAC cells samples by standard well-known laboratory protocols. The activity of xanthine oxidase (XOD) was determined following the Bergmayer method (25), catalase (CAT) according to Beers and Sizer (26), peroxidase (Px) according to Simon *et al*. (1974) (27), glutathione peroxidase (GSHPx) according to Beuthler [28] and glutathione reductase (GR) according to Goldberg and Spooner (20). The quantity of reduced glutathione (GSH) (non-protein SH) was also determined as well as the intensity of lipid peroxidation (LPx) using the Buege and Aust protocol (29).

*Statistical analysis*

 All analysis were run in triplicate and were expressed as mean ± standard deviation (SD). Student's *t*-test was carried out to identify statistical differences. *p* values of 0.05 or less (*p*<0.05) were considered to be statistically significant. Mean values between the groups in biochemical analyses were considered significantly different at *p*<0.05 confidence level, after the performance of the ANOVA single/double factor statistical analysis (30), followed by Bonferonni and Tuckey post-hoc tests.

## Results and discussion

*HPLC analysis of phenolic compounds*

 Major phenolics present in investigated raspberry pomace extracts from Meeker (ERM) and Willamette (ERW) cultivars were identified and quantified by HPLC analysis. Phenolic acids (protocatechuic, gallic, syringic, ellagic, caffeic, ferulic and synapic acid) and flavonoids (catechin, rutin, myricetin, luteolin and quercetin) were identified in ERM by matching their retention times (RT) and on-line ultraviolet (UV) spectra with those of standards. Additionaly, another phenolic acids (syringic and caffeic acids) were identified in ERW. It should be noted that catechin (flavan-3-ol) was detected at relatively high levels (103.07 mg/100g in ERW and 50.2 mg/100g in ERM). The content of total and individual phenolic compounds, quantified at 280, 330 or 360 nm, depending on their max response, is listed in Table 1. The total phenolic content is higher in ERW (410.66 mg per 100 g pomace) than in ERM (338.80 mg per 100 g pomace). Protocatechuic acid was found to be the main phenolic acid in raspberry pomace extracts from Meeker (215.28 mg/100g) and Willamette (214.47 mg/100g) cultivars. Both investigated extracts showed a high levels of gallic acid varying from 63.44 mg/100g in ERM to 77.87 mg/100g in ERW. Among the flavonols present in investigated raspberry pomace extracts, rutin was predominant. 

**Table 1:** Distribution of polyphenols in raspberry pomaces from Meeker (ERM) and Willamette (ERW) cultivars

**Peak**	**Compound**	**ERM**	**ERW**
1	Protocatechuic acid	215.28 ± 8.56	214.47 ± 10.07
2	Gallic acid	63.44 ± 2.19	77.87 ± 2.96
3	Syringic acid	n.d.	2.58 ± 0.10
4	Ellagic acid	1.16 ± 0.05	4.55 ± 0.18
5	Caffeic acid	n.d.	0.35 ± 0.01
6	Ferulic acid	2.04 ± 0.09	0.19 ± 0.01
7	Synapic acid	0.57 ± 0.02	0.47 ± 0.02
8	Catechin	50.2 ± 2.50	103.07 ± 4.26
9	Rutin	5.14 ± 0.16	5.79 ± 0.26
10	Myricetin	0.44 ± 0.02	1.02 ± 0.04
11	Luteolin	0.14 ± 0.01	0.09 ± 0.01
12	Quercetin	0.39 ± 0.02	0.21 ± 0.01
	Total	338.80	410.66

Data expressed as mg per 100 g of pomace and presented as mean ± SD

The major compounds found in the raspberries extracts and responsible for their radical scavenging activity have been described in literature and include phenolic acids as caffeic, ellagic, ferulic, *p*-cumaric, *p*-hydroxybenzoic and chlorogenic (31), flavan-3-ol (11), flavonol as catechin and epicatechin (32) and flavonoids as quercetin and myricetin (33). But, after juice processing, the most of phenols are present in pomace (16), and the observed antioxidant and cytotoxic activities is also due to a synergistic action of the different compounds present. The composition of the plant extract depends on many factors, such as the state of the original plant (variety, plant part and maturation stage), the geographical origin, climate conditions, harvesting date and storage, and also other environmental factors, and processing techniques.

*Radical scavenging activities*

The free radical scavenging activity of ERM and ERW was evaluated on different free radical species: stable 2,2-diphenyl-1-picrylhydrazyl radical (DPPH^•^) and reactive hydroxyl radical using ESR spectroscopy. 

 Stable DPPH radicals have been used to evaluate the radical scavenging ability of the investigated extracts. In all cases, a typical ESR spectrum of DPPH radicals, with five lines of relative intensities 1:2:3:2:1 and hyperfine splitting constant *a*_N_ = 9.03, was observed (34). The H-transfer reactions from antioxidants, present in ERM and ERW, to DPPH^•^ were monitored by recording the decay of the DPPH^•^ ESR signal. The present investigation has shown that ERM and ERW cultivars exhibited DPPH radical scavenging activity in a concentration dependant manner and the results were compared with the standard compound, indicating their abilities to act as radical scavengers.

**Figure 1:**
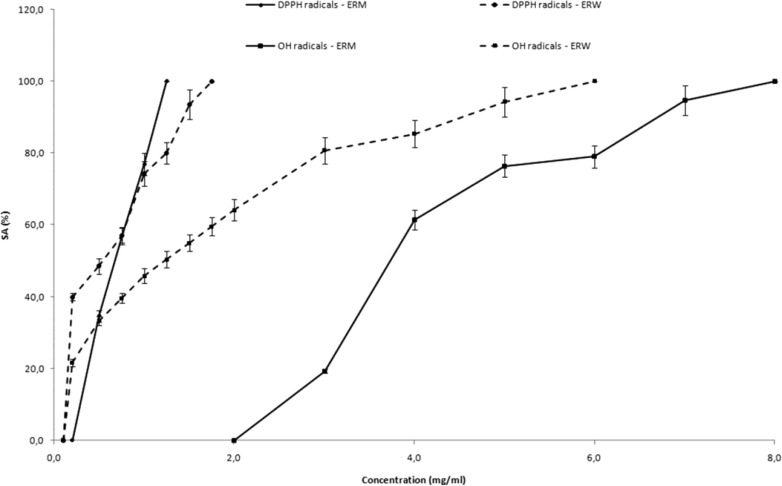
Scavenging activity of different concentrations of ERM and ERW on the a) DPPH radicals (SA_DPPH_^•^) and b) DMPO-OH spin adduct during the Fenton reaction (SA^.^_OH_) Data are given as inhibition percentages of DPPH radical or DMPO-OH spin adduct peak height measured by ESR and are mean   SD of three independent experiments

The DPPH radical scavenging activity of ERM and ERW increased with the increase of concentration. Complete scavenging of DPPH radicals (SA_DPPH_^•^ = 100%) was reached at the concentration of 1.25 mg/mL of ERM and 1.75 mg/mL of ERW. The SA_DPPH_^•^ of different concentrations of raspberry pomace extracts are presented in Figure1. 

Hydroxyl radicals are major reactive oxygen species inducing biological damage and lipid peroxidation. As hydroxyl (^•^OH) free radicals are highly reactive, with relatively short half-lives, the concentrations found in natural systems are usually inadequate for direct detection by ESR spectroscopy. Spin-trapping is a chemical reaction that provides an approach to help overcome this problem. These radicals are identified because of their ability to form nitroxide adducts (stable free radicals form). Hydroxyl radicals generated by the Fenton reaction (Fe^2+^/H_2_O_2_) were trapped by DMPO, forming DMPO-OH spin adducts which were detectable by ESR. In all cases the typical ESR spectra of DMPO-OH spin adduct characterized by quartet of lines with relative intensities 1:2:2:1 and hyperfine splitting constant a_N_=a_H_=14.9 G was noticed. The hydroxyl radical scavenging activity of ERM and ERW increased with the increase of concentration (Figure 1). Complete elimination of the hydroxyl radical is achieved at higher concentrations (8.0 mg/mL of ERM and 6.0 mg/mL of ERW).

 The SA_DPPH_^•^ and SA^•^_OH_ values of raspberry pomace extracts were determined based on radical scavenging activities of raspberry pomace extracts. The IC_50_^DPPH•^ varied from 0.67 for ERM to 0.54 mg/mL for ERW, while the IC_50_^•OH^ values varied from 3.73 for ERM to 1.23 mg/mL for ERW. 

It was observed that all investigated raspberry pomaces were very effective on DPPH^•^ than hydroxyl radicals and ERW was recognized as the most effective. In comparison to BHA as the positive control (IC_50_^DPPH^^•^ = 0.03 mg/mL; IC_50_^•OH ^= 1.51 mg/mL), both raspberry extracts exhibited affectual free radical activity.

The antioxidant activity of phenolic compounds is due to their ability to scavenge free radicals, donate hydrogen atoms or electron, or chelate metal cations. The structure of phenolic compounds is a key determinant of their radical scavenging and metal chelating activity, and this is referred to as structure–activity relationships (36).

*Cytotoxic activity against Ehrlich’s tumour in mice*

**Figure 2:**
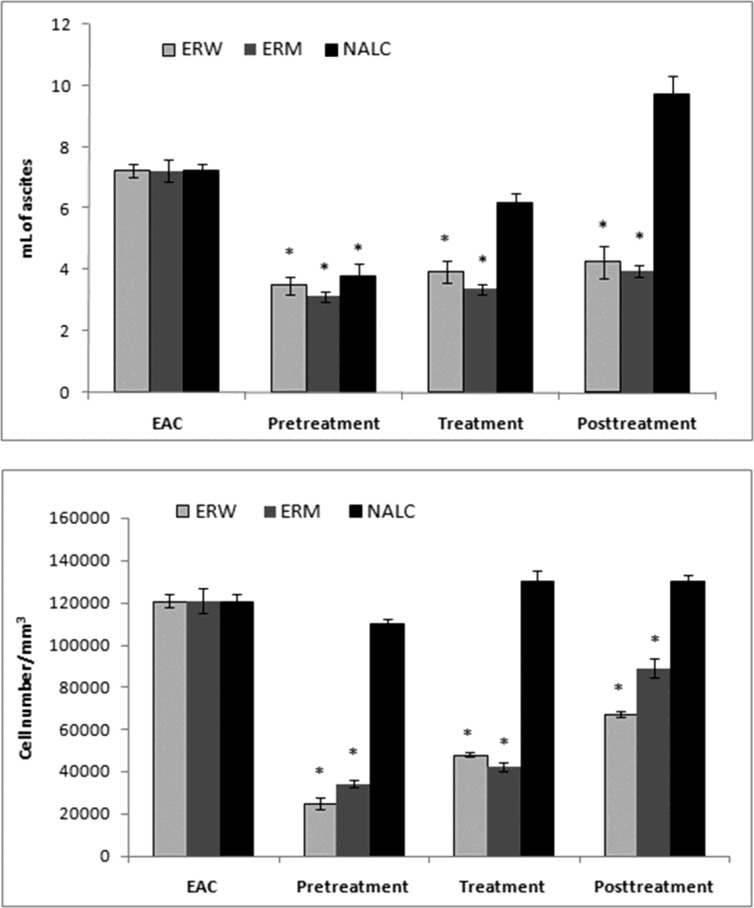
Effects of 2 mg/kg (i.p.) of ERW, ERM and N-acetyl-L-cysteine on (a) EAC ascites volume and (b) EAC cell number. EAC – untreated control;* Significantly different from the EAC group at p < 0.05 (compared with untreated control

**Figure 3:**
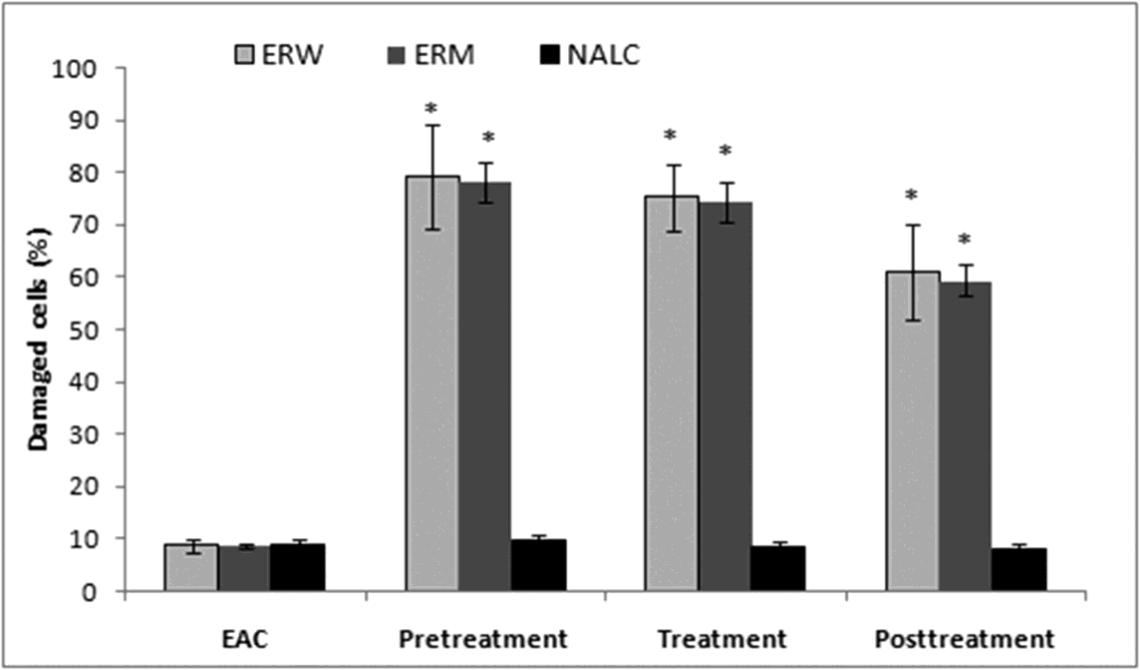
Effects of 2 mg/kg (i.p.) of ERW, ERM and N-acetyl-L-cysteine on EAC cell viability. EAC – untreated control; * Significantly different from the EAC group at p < 0.05 (compared with untreated control

The Ehrlich ascites tumor cell (EAC) is a spontaneous murine mammary adenocarcinoma  adapted to ascites form  and carried in outbred mice by serial intraperitoneal (i.p.) passage. It is a cancer of epithelial tissue that has glandular origin, glandular characteristics or both. It is a rapidly growing carcinoma with very aggressive in cell proliferation and is able to growin almost all strains of mice. In ascetic form, it has been used as a transplantable tumor model to investigate the antitumor effect of various substances (24). In the present study, we investigated antitumour properties of ERM and ERW methanolic extracts assessed by parameters such as ascites volume, tumour cell count and cell viability; the results are shown in (Figure 2, 3). The preliminary results using different doses (0.2, 0.5, 1.0, 1.5 and 2.0 mL/kg b.w.) indicated that 2.0 mg/kg b.w. (intraperitoneally) was the most effective dose. 

*Number of EAC cells and cell viability.* EAC cells were implanted into mice that were either pretreated, treated at the time or posttreated with the ERW or ERM. A significant decrease in the EAC ascites volume was observed in mice pretreated with both ERW and ERM (Figure 2a). There were no significant differences between the extracts, although the tumour growth inhibition was stronger with ERM. On the other side, 2 mL/kg b.w. of NALC as a proven antioxidant significantly inhibited the EAC ascites volume growth in pretreated mice, and applied as a posttreatment, even promoted the ascites growth. The EAC cell numbers were decreased in all extract-treated groups compared with the untreated EAC control group (Figure 2b). The largest decreases in EAC cell numbers were observed in the pretreated mice. Furthermore, we observed a decreased EAC cell viability after administration of the ERW or ERM (Figure 3). It can be noticed that the pretreatment showed better activity than treatment and posttreatment with ERW and ERM. 

Activities of the antioxidant enzymes (SOD, CAT, Px, GSHPx, GR), as well as the amount of GSH and intensity of LPx in the EAC cells are shown in Table 2. Application of extracts alone did not affect the antioxidant status of healthy animals and levels of examined antioxidant enzymatic systems. On the other hand, the antioxidant enzymes activities were significantly different in EAC groups pretreated and treated with ERW or ERM in comparison to untreated EAC control group. The activity of XOD was significantly increased, while the CAT activity was significantly decreased in pretreated groups as well as the activity of Px. The activity of enzyme complex GR and GSHPx, involved in the regeneration of GSH, was significantly increased (3-fold and 4.6-fold respectively) in pretreated groups, thus indicating that the induction of oxidative stress may play an important role in antitumour properties of a certain bioactive compounds. This was also in accordance with the results obtained for the measured amount of GSH. Glutathione (GSH) plays a crucial role as an endogenous antioxidant system. Particularly, high concentrations were found in liver and GSH is known to have a key function in protective processes (37, 38). The levels of GSH were high in EAC mice indicating the low level of oxidative stress in tumour cells. Treatment with ERW or ERM was found to deplete the GSH content compared to EAC control animals, which is probably due to its utilization by an excessive amount of free radicals. The oxidative stress may lead to significant damage of the macromolecules such as lipids and can induce lipid peroxidation *in vivo*. The hypothesis which claims that oxidative stress is involved in tumour cells apoptosis/necrosis was also checked by measuring the levels of cell membrane oxidative damage (LPx). The malonyldialdehyde (MDA) content (as indicator of LPx EAC cell membrane damage) in ERW and ERM - pretreated mice was significantly increased in comparison to the untreated EAC control group. 

The majority of polyphenols present in raspberry are flavonoids and phenolic acids that are an integral part of the human diet. There is a number of studies indicating the beneficial effects of berries extracts and isolated constituents on oxidative and other cellular processes leading to cancer development (39, 40). Laboratory rodent studies have shown that polyphenols have cancer-preventing properties and considered to be potential chemopreventive agents (41-43). They can influence important cellular and molecular mechanisms associated with multiple carcinogenic steps, such as expression of key proteins in signal transduction pathways (e.g., mitogen activated protein kinases (MAPKs) or activator protein (AP)-1), the transcription factor nuclear factor-kappa B (NF-*κ*B) and its downstream gene products, modulation of cell-cycle regulation, and induction of apoptosis (43), which affect cell differentiation, proliferation and apoptosis, immune responses and metabolism of carcinogens (44). Also, catechin has been found to inhibit PI3K/Akt activation, resulting in the modulation of Bcl-2 family proteins and leading to the enhanced apoptosis of bladder cancer cells (45). But, under oxidative stress, redox-dependent proteins are modified, redox signalling pathways are disrupted, and peroxides and free radicals are produced, leading to damage of cell components, including proteins, lipids, and DNA.

**Table 2:** Effects of ERW and ERM on the activities of antioxidant enzymes, glutathione levels and intensity of lipid peroxidation in EAC cells

**Group**	**XOD**	**CAT**	**Px**	**GR**	**GSHPx**	**GSH**	**LPx**
EAC	0.152±0.013	0.512±0.023	0.328±0.001	2.24±0.05	0.783±0.011	1.63±0.01	0.031±0.001
EAC +ERW pretreatment	3.71±0.21*	0.344±0.095*	0.887±0.02*	3.03±0.25	2.34±0.54*	1.44±0.02	0.15±0.010*
EAC+ERW treatment	0.135±0.043*	0.458±0.076	0.214±0.014	1.22±0.07	0.772±0.12	1.51±0.06	0.042±0.007
EAC+ERW posttreatment	0.105±0.021	0.579±0.047	0.147±0.018	1.15±0.11*	0.615±0.08	1.627±0.01	0.030±0.005
EAC +ERM pretreatment	3.88±0.35*	0.304±0.056*	0.912±0.05*	2.64±0.31	3.07±0.61*	1.36±0.04	0.14±0.015*
EAC+ERM treatment	0.228±0.045*	0.402±0.055	0.183±0.023	1.01±0.09	0.716±0.14	1.59±0.08	0.035±0.005
EAC+ERM posttreatment	0.125±0.017	0.447±0.063	0.159±0.024	1.48±0.17*	0.594±0.05	1.65±0.11	0.025±0.009

Values are expressed as means ± SD of six mice at *p*<0.05

Activities of xanthine oxidase (XOD), catalase (CAT), peroxidase (Px), glutathione reductase (GR), glutathione peroxidase (GSHPx) are expressed in µmol/mL EAC cells.

Amount of reduced glutathione (GSH) is expressed in nmol GSH/mL EAC cells

Intensity of lipid peroxidation (LPx) is expressed in nmol MDA/mL EAC cells.

* Significantly different from the EAC group at *p*<0.05

## Conclusion

We conclude that raspberry pomace extracts may be considered as a good source of natural compounds, especially phenolic acids and flavonoids with significant antioxidant activity by effectively scavenging DPPH and hydroxyl radicals. The extracts also exhibit cytotoxic properties against Ehrlich carcinoma (EAC) cells *in-vivo* and play a role as a cancer-preventing agent. These findings indicate that raspberry pomace extracts should be regarded as potential nutraceutic resource and used as functional food ingredient. Still, more researches elucidate the possible mechanism of action. 
